# Truth Distancing? Whistleblowing as Remedy to Censorship during COVID-19

**DOI:** 10.1017/err.2020.49

**Published:** 2020-05-06

**Authors:** Vigjilenca ABAZI

**Affiliations:** * Maastricht University; email: v.abazi@maastrichtuniversity.nl.

## Abstract

In the COVID-19 pandemic, whistleblowers have become the essential watchdogs disrupting suppression and control of information. Many governments have intentionally not disclosed information or failed to do so in a timely manner, misled the public or even promoted false beliefs. Fierce public interest defenders are pushing back against this censorship. Dr Fen and Dr Wenliang were the first whistleblowers in China to report that a new pandemic was possibly underway, and ever since, numerous other whistleblowers around the world have been reporting on the spread of the virus, the lack of medical equipment and other information of public interest. This paper maps the relevant whistleblowing cases in China, the USA and Europe and shows that many whistleblowers are initially censored and face disciplinary measures or even dismissals. At the same time, whistleblowing during the COVID-19 pandemic has drawn public attention to the shortcomings of institutional reporting systems and a wider appreciation of whistleblowers as uniquely placed to expose risk at early stages. Ultimately, whistleblowing as a means of transparency is not only becoming ever less controversial, but during COVID-19 it has become the “remedy” to censorship.

## I. A pandemic in a post-factual world

How do governments deal with a pandemic in a “post-factual” world? Far too many have not disclosed information or failed to do so in a timely manner, misled the public or even promoted false beliefs. Transparency has been particularly underserved by those leaders who generally tend to be dismissive of truth and facts. More than error or miscalculation, censorship of information is at the core of how some governments and authorities manage this pandemic as they seek to control the narrative over its eruption and spread.

Whistleblowers have played a crucial role in exposing facts during the COVID-19 pandemic, starting with Dr Li Wenliang and Dr Ai Fen in China and numerous other (medical) workers around the world. Whistleblowers have disclosed information relevant for the spread of the virus, the lack of medical equipment and other information of public interest. Such information has been reported to the press, in social media as well as internally at their workplaces or through hotline calls to organisations working on the protection of whistleblowers.

This paper traces the main whistleblowing cases in China, the USA and Europe. In mapping these cases, this paper relies on data drawn from interviews with stakeholders and experts, news reports and reports by organisations working with whistleblowers, and, where possible, on official government documents. At the time of writing, we are still in the midst of the COVID-19 crisis and therefore we are yet to receive further official reports and analysis. In this respect, this paper offers an initial tentative analysis of the current developments, with the aim of discerning some broader patterns from the available material.

The paper observes that many whistleblowers are initially censored and face disciplinary measures or even dismissals, although there are important differences between China and the USA/Europe on the level of censorship and information control. Whilst backlash against whistleblowers is not news,^1^ whistleblowing during the COVID-19 pandemic is impacting the public opinion on the acceptability of whistleblowers and has mobilised civil society to increase cooperation globally in acting as a critical watchdog on government censorship of whistleblowers. The paper concludes that censorship is challenged by many fierce public interest defenders who confront information control and expose relevant facts about the COVID-19 pandemic.

## II. “Truth and rumour”: censorship and Chinese whistleblowing law

“I regret that back then I didn’t keep screaming out at the top of my voice.”

Dr Ai Fen, Wuhan Central Hospital^2^

In December 2019, Dr Li Wenliang posted a message to a social media chat group, which included other medical doctors, about some patients showing signs of a new illness similar to severe acute respiratory syndrome (SARS). His communication with his colleagues soon reached the local authorities. On 3 January 2020, Dr Wenliang was detained for “spreading false rumours” and was forced to sign a police document admitting that he had “seriously disrupted social order”.^3^ Article 41 of the Chinese Constitution foresees the “right to criticize and make suggestions to any state organ or functionary” and to raise complaints and charges against, or exposures of, any state organ or functionary for violation of the law or negligence of duty. However, “fabrication or distortion of facts for the purpose of libel or frame-up” is prohibited. Therefore, during the reporting process, it is essential to assess whether a report may be categorised as a “rumour”. Chinese law does not define the meaning of a “rumour”, although many regulations foresee penalties for “spreading rumours”.^4^

Quite contrary to disrupting social order, Dr Wenliang’s attempts to alert his colleagues and later the public were crucial to disrupting information suppression and control.^5^ This kind of “disruption” is essential for generating public knowledge, and such disclosure of information by insiders is necessary for the purposes of accountability.^6^ Dr Wenliang blew the whistle that a new pandemic was possibly underway, and he was not alone in raising the alert.^7^ It is reported that on the same day as Dr Wenliang’s post in the chat group, another medical doctor, Dr Ai Fen, had reported to a hospital’s public health department and infection department that she had seen a test sheet mentioning symptoms of SARS.^8^ Hospitals, however, may defer to local health authorities about reporting infections, apparently to avoid surprising and embarrassing local leaders.^9^ The mayor of Wuhan publically admitted that the news about the virus should have been made known earlier and acknowledged that, in his role as mayor, he could only release information upon receiving authorisation from the relevant authorities.^10^

COVID-19 has brought the discussion on whistleblower protection to the fore in China, and a wider public debate has ensued as to the value of whistleblowers. The censorship of information about this pandemic and the responses of authorities bear a similarity to the case of Shuping Wang, who blew the whistle in the 1990s exposing the handling of HIV and hepatitis epidemics in China.^11^ Yet unlike before, public opinion is shifting in terms of the role of the whistleblower. Legally, a whistleblower in China is viewed as an enforcer of government regulation.^12^ Current Chinese law on whistleblowing offers protection in bits and pieces with provisions in the criminal law procedure, labour law, work safety, foods and drugs, product quality, securities and financial fields. A Chinese legal expert explains that “the focus of the policy is to encourage and protect insiders who have real inside information to come forward to assist the government in performing its regulatory functions”.^13^

For the Chinese government, whistleblowing is viewed as a social control mechanism, especially for controlling corruption of officials.^14^ The COVID-19 whistleblowers in China, however, do not fit this narrow view of a whistleblower as an agent who enforces regulation on behalf of the government. Rather, they are pointing to a role of a whistleblower who raises an alarm in the public interest, even when the government seeks to supress such information or control its distribution. Dr Wenliang’s case illustrates this point. When he died from COVID-19 in February 2020, it sparked widespread public anger, with many citizens openly expressing calls for freedom of speech in social media (leading to nearly two million views) that were later censored, and the phrase “#Wuhan government owes Dr Li Wenliang an apology” received tens of thousands of views before it was removed.^15^ Hence, in Chinese public opinion, Dr Wenliang is seen as a whistleblower who sought to raise an alarm in the public interest and whose voice was being silenced rather than supported by the government. This in turn highlights that the interest of the people to know can be in tension with the government’s efforts to withhold that information. The COVID-19 pandemic has drawn public attention in China to the role of whistleblowers as voices of public interest and to whistleblowing as a form of freedom of expression. Whether such a view will be sustained or even transposed into law remains to be seen.

## III. Censorship and silencing whistleblowers in the USA and Europe

Whilst the effects of supressing and controlling information in China are severe, workers around the globe, especially medical workers at the frontline of fighting COVID-19, have faced pressure from governments and authorities to remain silent. As *The New York Times* reported:

In New York City, the epicenter of the crisis in the United States, every major private hospital system has sent memos in recent weeks ordering workers not to speak with the media, as have some public hospitals.^16^

In addition to constraining their freedom to speak out on the COVID-19 pandemic, workers have also experienced disciplinary measures and even dismissals in instances where they have expressed concerns about their working conditions. In the USA, the Government Accountability Project, a whistleblower protection and advocacy organisation based in Washington, DC, has reported numerous cases of whistleblowers being fired for speaking out. For example, in Seattle, an emergency room physician was fired after giving an interview to a newspaper about inadequate protective equipment and testing, as was a nurse in Chicago for asking for better equipment in emails with her colleagues.^17^

On the other side of the Atlantic, the situation has been similar. In the UK, medical professionals of the National Health Service have been put under pressure not to speak out, and media report that tactics for silencing workers include “threatening emails, the possibility of disciplinary action, and some people even being sent home from work”.^18^

In European Union (EU) countries where the rule of law had deteriorated even prior to COVID-19,^19^ even harsher pressures have been reported. For example, upon using social media to alert on missing masks and equipment, a healthcare professional in Poland was fired by their hospital director.^20^ This situation led to a reaction by Poland’s Ombudsman to the Ministry of Health, asking that the decision to fire the health worker be revoked and reminding the Ministry of the constitutional freedoms and rights for freedom of expression in Poland.^21^ The issue is not isolated to one doctor, but seems to have become a growing practice, with medical staff being asked to consult with management and forbidding doctors to discuss their work or matters pertaining to COVID-19 directly with journalists.^22^ Hungary can be seen as an even more extreme case, since blowing the whistle is not even a possibility due to measures limiting freedom of expression that are directly targeted at journalists, including a prison term of five years for “fake” reporting.^23^

Pressures on whistleblowers and freedom of speech have also been reported in Serbia, an EU candidate state. For example, a whistleblower sought to reveal that the union of workers is charging for masks that medical staff should use,^24^ or in another case a local TV crew staffer was arrested after investigating a tip from a whistleblower^25^ under the alleged accusation that the reporter did not respect the precautionary health measures for COVID-19 when he entered the city hall.

Against this background of pressure and dismissals of whistleblowers and even possible imprisonment of journalists who help bring these facts to the public, more than 95 civil organisations across Europe and globally have come together to issue a statement calling for the protection of whistleblowers and making it clear that they, as a civil society, will continue to monitor and expose the censorship of whistleblowers.^26^ Organisations such as Protect in the UK offer specific advice lines for COVID-19 whistleblowers and information for workers as well as members of vulnerable groups.^27^ Transparency International Ireland has also published new guidance on whistleblowing for workers and guidance for employers during the COVID-19 pandemic.^28^ The guidance includes advice on reporting COVID-19-related concerns to employers, regulators or the media, as well as measures employers can take to respond to concerns effectively. In addition to the hands-on work with guiding and helping whistleblowers, civil society is also focused on advocating for turning these solutions into a coherent policy. As explained by Anna Myers, the Executive Director of Whistleblowing International Network (WIN), a crisis like the COVID-19 pandemic reveals that: “Everyone, not just the appointed decision-makers, but everyone, everywhere needs information in order to make informed decisions”.^29^

This aspect of COVID-19 whistleblowing is having an impact on public opinion about who may be a whistleblower, why it is important to protect individuals who speak out as well as why whistleblowing can be a unique tool for channelling information to the public. Trying to make use of this favourable public opinion on whistleblowing, civil organisations are connecting and mobilising globally and monitoring how governments and businesses are responding and whether they are trying to stop or punish those who are blowing the whistle. For example, WIN is gathering this information to set up a COVID-19 Whistleblowing Information Hub that would be used for current work on protecting whistleblowers, but also for future advocacy for advancing protections.^30^ In other words, the appreciation for information sharing during this pandemic has opened up new ground for showing the value of whistleblowing and possibly transposing that public support into a longer-lasting effect by establishing and expanding legal protections or, where they exist, to ensuring that they are being adequately implemented.

## IV. COVID-19 watchdogs

Whistleblowers as watchdogs during the COVID-19 pandemic have put in full display the shortcomings of institutional reporting systems, as well as the distinct value of whistleblowing in exposing risk at early stages. Lack of transparency and information-sharing failures have been systematic and global during the COVID-19 pandemic. Censorship of information by governments and authorities indicates an inclination to put reputational interests ahead of solving serious problems such as shortages of medical equipment and the work safety of workers, particularly medical professionals. Censorship only enables governments to control the narrative and public opinion in the short term. When dealing with a crisis such as COVID-19, sustaining transparency is not only a checklist item for good governance; it can actually save lives. Whistleblowers are filling this transparency gap and have become an essential watchdog for keeping governments in check in terms of how they manage this pandemic. The cases discussed in this paper show that the purpose of these whistleblowers has been to expose serious errors or the lack of resources in the health system in order to ensure that errors are rectified as soon as possible. At the same time, these whistleblowers have been pressured to remain silent and disciplined or dismissed in cases when they have spoken out. In extreme cases, the journalists who sought to expose their stories have faced threats of imprisonment. Such efforts to curtail freedom of speech have invigorated civil society to mobilise globally in advancing long-term policy solutions and protections for whistleblowers. Ultimately, whistleblowing as a means of transparency is not only becoming ever less controversial, but during COVID-19, it has become the “remedy” to censorship.

## Footnotes

1 See T Muller, *Crisis of Conscience: Whistleblowing in an Age of Fraud* (New York, Penguin Random House LLC 2019); W Vandekerckhove, C James and F West, “Whistleblowing: The Inside Story – A Study of the Experiences of 1,000 Whistleblowers”, Project Report, Public Concern at Work, UK, 2013; the Council of Europe report on “The protection of ‘whistleblowers’ in 2009” (see P Omtzigt, Committee on Legal Affairs and Human Rights, “The Protection of Whistle-Blowers”, Report, Doc. 12006, 29 September 2009); Report of the UN Special Rapporteur to the General Assembly on the Protection of Sources and Whistleblowing in 2015; but for recent legal protections in Europe, see V Abazi, “Whistleblowing in Europe: A New Era of Legal Protections” in P Czech, L Heschl, K Lukas, M Nowak and G Oberleitner (eds), *European Yearbook on Human Rights* (Cambridge, Intersentia 2019) pp 91–111.

2 As quoted in SL Myers, “China Created a Fail-Safe System to Track Contagions. It Failed”, *The New York Times*, 29 March 2020 <https://nyti.ms/2YnlwEH> (last accessed 30 April 2020).

3 H Davidson, “Chinese Inquiry Exonerates Coronavirus Whistleblower Doctor”, *The Guardian*, 20 March 2020 <https://bit.ly/2VSh8Mt> (last accessed 30 April 2020).

4 Eg Art 25 of the Law on Public Security Administration Punishments; Arts 105 and 221 Criminal Law; Art 229 can be up to seven years in prison. See W Junqi, “Truth, Rumors, and the ‘whistleblower’ System”, 19 February 2020, original source in Chinese <https://bit.ly/2KLO3vF> (last accessed 30 April 2020).

5 Chinese authorities have acted on many fronts to control the narrative on the origin of the pandemic and its spread and China’s handling of the pandemic, including censorship of academic research as well as expelling journalists from *The New York Times*, *The Wall Street Journal* and *The Washington Post*; see, respectively, S Kirchgaessner, E Graham-Harrison and L Kuo “China Clamping Down on Coronavirus Research, Deleted Pages Suggest”, *The Guardian*, 11 April 2020 <https://bit.ly/2YkzAPq> (last accessed 30 April 2020); K Manson and Y Yang, “China Expels Journalists from Multiple US Media Groups”, *Financial Times*, 17 March 2020 <https://on.ft.com/2WjxEUO> (last accessed 30 April 2020).

6 V Abazi, “Democracy and Disruption”, Yale Law School Research Paper, 2019.

7 For a view that Dr Wenliang cannot be categorised as a whistleblower as he did not follow internal reporting procedures, see “Why Is Li Wenliang Not a ‘whistleblower’?” 9 February 2020, original source in Chinese: <http://www.jintiankansha.me/t/Neisunzuil> (last accessed 30 April 2020).

8 G Jingqi, “Whistleblower”, 10 March 2020, original source in Chinese, available in archived form only <http://archive.is/OLdHs>.

9 Myers, supra, note 2.

10 Al Jazeera, “China: Truth in a Pandemic”, 10 April 2020 <https://bit.ly/3d0XSSv> (last accessed 30 April 2020). Chinese law requires the disclosure of information with authorisation as provided in Art 19 of the “Regulations on the Disclosure of Government Information”. See details on how Wuhan did not handle the disclosure of information in accordance with law in an interview with Professor Mok Yu-Chuan, China Law Society Research Association of Administrative Law, Xinhua News Agency, “Discussion on Whistleblower System and Other Related Issues to the Prevention and Control of Epidemics”, 7 February 2020, original source in Chinese <http://www.law.ruc.edu.cn/article/?id=55769> (last accessed 30 April 2020).

11 BBC News, “Shuping Wang: Whistleblower Who Exposed HIV Scandal in China Dies”, 26 September 2019 <https://www.bbc.com/news/world-asia-49834904> (last accessed 30 April 2020).

12 L Huihui, “Reflections on the ‘Whistleblower’ System”, *China Law Insight, King & Woods Mallesons*, 18 October 2019, original source in Chinese <https://bit.ly/2USVQML> (last accessed 30 April 2020).

13 Supra, note 7.

14 T Gong, “Whistleblowing: What Does It Mean in China?” (2000) 23(11) International Journal of Public Administration 1899.

15 V Yu, “Hero Who Told the Truth: Chinese Rage over Coronavirus Death of Whistleblower Doctor”, *The Guardian*, 7 February 2020 <https://bit.ly/2KP3O4V> (last accessed 30 April 2020).

16 N Scheiber and BM Rosenthal, “Nurses and Doctors Speaking Out on Safety Now Risk Their Job”, *The New York Times*, 9 April 2020 <https://nyti.ms/3aQwJjN> (last accessed 30 April 2020).

17 See S Feinstein, “COVID-19: The Largest Attack on Whistleblowers in the World”, *Government Accountability Project*, 8 April 2020 <https://bit.ly/3bTgX98> (last accessed 30 April 2020).

18 S Johnson, “NHS Staff Forbidden from Speaking Out Publically about Coronavirus”, *The Guardian*, 9 April 2020 <https://bit.ly/3bUjXCc> (last accessed 30 April 2020).

19 See L Pech and KL Scheppele, “Illiberalism Within: Rule of Law Backsliding in the EU” (2017) 19 Cambridge Yearbook of European Legal Studies 3.

20 A Piton, “The Hospital Director Fired the Midwife Because She Alerted on Facebook That Masks and Equipment Were Missing”, *Wyborcza.pl*, 24 March 2020, original source in Polish <https://bit.ly/3d27Fru> (last accessed 30 April 2020).

21 Letter by the Ombudsman to the Minister of Health <https://bit.ly/2z3n4cc> (last accessed 30 April 2020).

22 K Nowakowska, “Doctors Are Silent about Coronavirus. Doctors Silence Medics”, *Gazeta Prawna*, 26 March 2020, original source in Polish <https://bit.ly/3f88aCo> (last accessed 30 April 2020).

23 European Federation of Journalists, “COVID-19: Journalists Threatened with Imprisonment in Hungary”, 23 March 2020 <https://bit.ly/2z1iBqw> (last accessed 30 April 2020).

24 AS and MJ, “Union Charges Employees for Protective Masks, Including an Administrative Ban”, *Južne Vesti*, 30 March 2020, original source in Serbian <https://bit.ly/2VQpKDe> (last accessed 30 April 2020).

25 KTV Zrenjanin, “Our Team Arrested on a False Accusation That They Did Not Disinfect”, *N1*, 26 March 2020, original source in Serbian <https://bit.ly/2VWBLap> (last accessed 30 April 2020).

26 The Good Lobby, “Coalition to Make Whistleblowing Safe during COVID-19 and Beyond”, 6 April 2020 <https://bit.ly/2KOccSl> (last accessed 30 April 2020).

27 See Protect information <https://bit.ly/3bTU3OX> (last accessed 30 April 2020).

28 See Transparency International Ireland <https://bit.ly/35na914> (last accessed 30 April 2020).

29 Interview with Anna Myers, 21 April 2020, on file with the author.

30 ibid.

